# Defectors Can Create Conditions That Rescue Cooperation

**DOI:** 10.1371/journal.pcbi.1004645

**Published:** 2015-12-21

**Authors:** Adam James Waite, Caroline Cannistra, Wenying Shou

**Affiliations:** 1 Molecular and Cellular Biology Program, University of Washington, Seattle, Washington, United States of America; 2 Division of Basic Sciences, Fred Hutchinson Cancer Research Center, Seattle, Washington, United States of America; Max-Planck-Institute for Evolutionary Biology, GERMANY

## Abstract

Cooperation based on the production of costly common goods is observed throughout nature. This is puzzling, as cooperation is vulnerable to exploitation by defectors which enjoy a fitness advantage by consuming the common good without contributing fairly. Depletion of the common good can lead to population collapse and the destruction of cooperation. However, population collapse implies small population size, which, in a structured population, is known to favor cooperation. This happens because small population size increases variability in cooperator frequency across different locations. Since individuals in cooperator-dominated locations (which are most likely cooperators) will grow more than those in defector-dominated locations (which are most likely defectors), cooperators can outgrow defectors globally despite defectors outgrowing cooperators in each location. This raises the possibility that defectors can lead to conditions that sometimes rescue cooperation from defector-induced destruction. We demonstrate multiple mechanisms through which this can occur, using an individual-based approach to model stochastic birth, death, migration, and mutation events. First, during defector-induced population collapse, defectors occasionally go extinct before cooperators by chance, which allows cooperators to grow. Second, empty locations, either preexisting or created by defector-induced population extinction, can favor cooperation because they allow cooperator but not defector migrants to grow. These factors lead to the counterintuitive result that the initial presence of defectors sometimes allows better survival of cooperation compared to when defectors are initially absent. Finally, we find that resource limitation, inducible by defectors, can select for mutations adaptive to resource limitation. When these mutations are initially present at low levels or continuously generated at a moderate rate, they can favor cooperation by further reducing local population size. We predict that in a structured population, small population sizes precipitated by defectors provide a “built-in” mechanism for the persistence of cooperation.

## Author Summary

When defectors appear in a population of cooperators due to mutation or migration, defectors should become more common because they exploit the resource provided by cooperators without making a fair contribution to its production. The environmental degradation caused by defectors eventually leads to population collapse, and should result in the extinction of cooperators and defectors alike. By explicitly tracking all individuals in a spatially-structured environment where they can migrate to different locations, we find that cooperation can survive due to under-appreciated consequences of defector-induced population collapse. Population collapse creates vacant territories which can support the growth of cooperator but not defector migrants. This leads to the counterintuitive result that cooperation sometimes survives better when defectors are initially present compared to when they are initially absent. When defectors are initially present, the emergence of mutants best adapted to defector-degraded environments can also promote cooperation. Thus, by reducing population size through physiological and evolutionary mechanisms, defectors create “built in” mechanisms that protect cooperation from defectors. This may help explain the near ubiquity of cooperation despite its inherent vulnerability.

## Introduction

A cooperator produces a common good (or benefit) available to all members of a population at a fitness cost to itself, and gains a net benefit if sufficiently reciprocated. However, cooperators are vulnerable to evolutionary displacement by defectors that exploit this benefit without paying the fitness cost of benefit production. Thus, the ubiquity of organisms displaying apparently cooperative behavior demands an explanation, and decades of experimental and theoretical research have been dedicated to unraveling this riddle.

Previous research has uncovered multiple mechanisms that promote cooperation [1,2]. Recognition allows cooperators to direct benefits to other cooperators and exclude or punish defectors [3]. In the absence of recognition mechanisms, a spatially-structured environment can facilitate cooperation by promoting repeated interactions between individuals. For example, during repeated interactions, cooperators can persist in, and even invade, defector-dominated populations if an individual can adjust its cooperating strategy based on memory of past experience. Examples include “tit-for-tat” [4] and “win-stay-lose-shift” [5]. Even when organisms cannot adjust their cooperating strategy, limited migration in a spatially-structured environment (“population viscosity”) means that cooperators will preferentially interact with their genetic relatives who are also likely to be cooperators (also known as “kin selection”) [6,7]. However, increased population viscosity also increases local competition between cooperators for non-cooperative resources, and therefore migration to sites with additional resource is necessary for cooperation to survive [8–12].

A group of spatially-separated populations connected through migration is known as a metapopulation. For cooperation to persist in a metapopulation, cooperator frequency must vary greatly among different populations. This is because, when cooperator frequency is uniform across populations, the cooperator frequency of the metapopulation will mirror that of each population and decline over time due to the advantage of defectors over cooperators. In contrast, when cooperator frequency varies across populations, individuals in a population dominated by cooperators (which are likely cooperators) will grow faster than individuals in a population dominated by defectors (which are likely defectors). Thus, the global frequency of cooperators can increase despite the fact that defectors are more fit than cooperators in individual populations. This phenomenon is described by the Price equation [13,14] and is also known as “Simpson's Paradox” [15,16]. A consequence of large variation in cooperator frequency is that population dynamics will be highly heterogeneous across the metapopulation: defector-dominated populations will go extinct while cooperator-dominated populations will thrive and migrate to populate vacant locations.

One mechanism that generates large variation in cooperator frequency is small population size. In the most extreme case, if populations are founded by a single individual, then each population will have a cooperator frequency of 1 (if the founder is a cooperator) or 0 (if the founder is a defector). The possibility of small population size promoting cooperation has been widely observed in simulations [17–22], and was also demonstrated in an experimental system using *Escherichia coli* that was periodically diluted to very low population size [23].

Ecologically-relevant ways to achieve small population size include the migration of a small number of individuals to a vacant space or exposure to harsh environments that only allow survival of a small number of individuals. The idea of harsh environments and small population size supporting the evolution of cooperation [24] has received theoretical [21,25,26] and experimental support, both in animals [27] and microbes [28–30].

Surprisingly, one obvious way to achieve small population size has been largely overlooked: defectors themselves. The way in which defectors feed back onto population dynamics can be complex and produce surprising outcomes. Experimentally, defectors can increase the total biomass of a population under some circumstances [31]. Previous theoretical work has shown that cooperation can survive defector invasion if defector-induced environmental degradation promotes a small level of migration in cooperators and defectors [32,33]. Defectors can also, in theory, support the long-term success of cooperation by maintaining selective pressure for partner recognition [34].

We reasoned that the small population size known to promote cooperation could be created by defectors. To test this idea, we investigated a model of microbial cooperation based on the production of a common good. As microbial populations often violate common theoretical assumptions such as weak selection, fixed group size, and linear relationships between cooperator frequency and fitness [35,36], we developed a model based on processes that have been experimentally observed or are measurable. Similar to previous approaches used to simulate community growth [37–39], our model combined stochastic and deterministic components. The stochastic, agent-based component tracked the birth, death, mutation, and migration of all individuals in a metapopulation. This allowed us to capture stochastic events at small population size that would have been missed by deterministic models. The deterministic differential equation component allowed us to efficiently model the amount of common good available to a population without tracking each individual molecule of common good.

We have found three ways in which defectors create conditions that rescue cooperation. First, during defector-induced population collapse, defectors occasionally go extinct before cooperators by chance, allowing cooperator-only but not defector-only populations to grow. Second, defectors cause population extinction, which creates empty locations that support cooperator but not defector migrants. This leads to the counterintuitive result that the initial presence of defectors in a population can sometimes improve the survival of cooperators when a subpopulation of cooperators mutate to defectors. Third, resource limitation, which can be precipitated by defectors, selects for rare cooperator and defector mutants adapted to this resource limitation. When defectors are initially present, this creates yet another population bottleneck beneficial to cooperation. These three factors act together to generate large variation in cooperator frequency across a metapopulation and therefore promote cooperation. Thus, by creating small population sizes, defectors provide a built-in mechanism that can rescue cooperation.

## Model

### Conceptual outline

**Fig 1:**
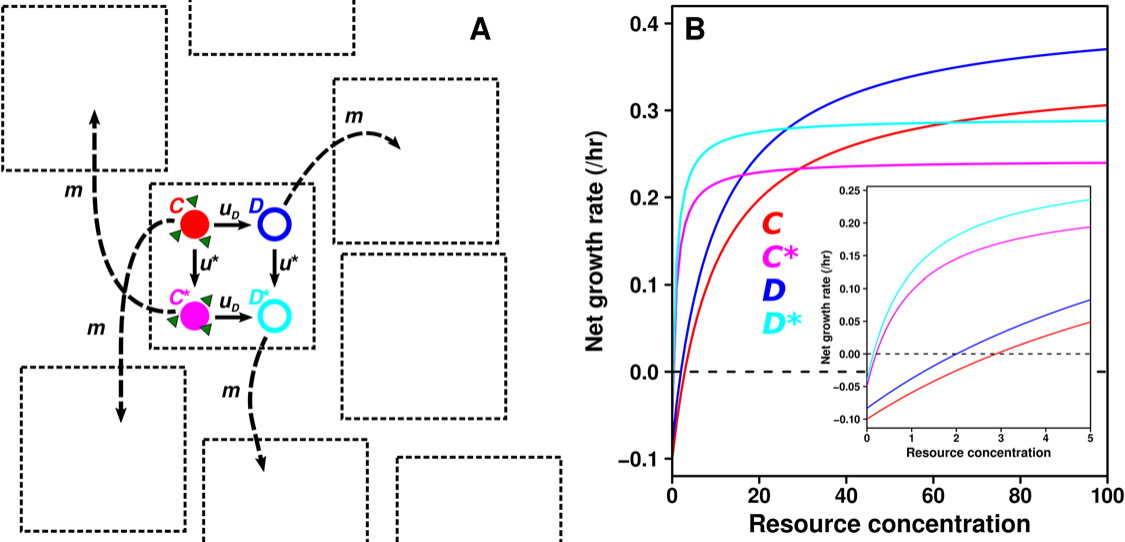
A social dilemma defined by resource use and fitness trade-offs. **A**) Model overview. Ancestor types mutate to evolved types (denoted by a '*') at rate u*. Cooperators (*C* and C*), which produce resource (green triangles) mutate at rate *u*_*D*_ to defectors (*D* and D*), which produce nothing. All types migrate randomly to new locations at rate *m*. **B**) When resource is abundant, *C* (red) and *D* (blue) have faster net growth rates than C* (magenta) and D* (cyan). When resource is low, C* and D* grow faster than *C* and *D*. However, the net growth rate of *D* is always greater than *C*, and the net growth rate of D* is always greater than C*, resulting in a social dilemma. (Inset) The fitness functions at low resource levels.

We modeled a social dilemma based on the production and consumption of common goods (Fig 1A). The cooperator type (*C*) generated publicly-available resource, which it also consumed in order to grow. The defector type (*D*) consumed this resource but did not produce it, and therefore enjoyed a fitness advantage which increased *D*'s birth rate and decreased its death rate relative to *C* (Fig 1B). Low-resource adapted mutant cooperators (C*) and defectors (D*), collectively referred to as “mutants”, grew much faster than their respective ancestor types in low-resource environments, and died more slowly in all environments (Fig 1B). To reflect experimental observations of fitness trade-offs [40–43], these mutants were poorly adapted to the resource-abundant environment of their ancestors (Fig 1B). This type of trade-off can arise, for example, when mutations increase surface expression of the transporter(s) responsible for resource uptake [42,44]. Increased transporter expression improves fitness in low-resource environments, but leads to excessive uptake and toxicity in high-resource environments [42,43]. Thus, the relative fitness of each type was ordered as *C* < *D* < C* < D* when resource was scarce and C* < D* < *C* < *D* when abundant (Fig 1B, see below for more details). When resource levels were intermediate, the fitness relationships changed at various line intersection points (Fig 1B); however, the fitness relationships always obeyed *C* < *D* and C* < D*.

Because mutations that reduce or abolish cooperative benefit naturally occur [45–47], each cooperator type mutated to its corresponding defector type at rate *u*_*D*_ (Fig 1A). We modeled cooperator to defector mutation but not the reverse, because the mutational target size for losing the ability to make common goods likely far exceeds that for regaining this ability. Mutation from ancestor to adaptive mutant occurred at rate u* (Fig 1A). In many simulations, we assumed that mutants adapted to low-resource environments and defectors were present at the beginning of simulations, because these variants are constantly generated from non-mutants and cooperators, respectively, during population growth [48].

We defined a *population* as a group of individuals residing in the same location, with each individual belonging to one of the four types (*C*, *D*, C*, or D*; Fig 1A). We defined a *metapopulation* as a set of populations that could interact through the migration of individuals (Fig 1A). Table 1 summarizes all parameter values used in this model.

**Table 1:** Parameter summary.

Parameters	Description	Value(s)
*i*	cooperator/defector index	0/1
*j*	ancestor/mutant index	0/1
*V*_00_	Maximum birth rate of *C* (hr^-1^)	0.450
*V*_10_	Maximum birth rate of *D* (hr^-1^)	0.500
*V*_01_	Maximum birth rate of C* (hr^-1^)	0.293
*V*_11_	Maximum birth rate of D* (hr^-1^)	0.333, 0.354
*K*	Resource giving half-maximal growth (units)	10
*d*	Death rate of *C* (hr^-1^)	0.1
*θ*	Mutant *K* advantage	10
*α*	Defector (mutant) death advantage	1.2 (1.2, 1.3)
*δ*	Mutant death advantage	2
*κ*	Population carrying capacity (individuals)	10^6^
*γ*	The amount of resource required to produce a new cell (units)	5.5
*β*	Maximum resource release rate (units/cooperator/hr)	2.4, 1.2, 0.6, 0.3, 0.15
*m*	Migration rate (hr^-1^)	0; 10^−4^–10^−12^ in 10-fold steps
*u*_*D*_	Cooperator to defector mutation rate (hr^-1^)	10^−7^
u*	Ancestor to evolved mutation rate (hr^-1^)	0; 10^−5^–10^−10^ in 10-fold steps
*Δt*	time step (hr)	0.005
	Number of locations	144

### Resource-dependent fitness functions

The birth rate of all types depended on the amount of resource available in their current location. We used a Holling Type II (or “Monod”) [49,50] function to relate resource concentration (*S*) to birth rate (*b*), because of its simplicity and empirical support. This function is defined by two parameters: *V*, the maximum birth rate, and *K* (sometimes referred to as the “Monod constant”), the concentration of *S* allowing half maximal birth rate. Let *i* = 0 or 1 denote cooperator or defector, respectively, and let *j* = 0 or 1 denote ancestor or mutant, respectively. Then the birth rate of a given type was described by

$$b_{ij}\left(S\right)=\frac{V_{ij}S}{K/\theta^{j}+S}\;,$$ (1)

where *θ* (>1) denoted the mutant *K* advantage over the ancestor when resource became limited due to mutant's higher affinity for resource.

Death was modeled as a separate process with a rate *d*_*ij*_ independent of *S*,

$$d_{ij}=\frac{d}{\alpha^{i}\delta^{j}}\;,$$ (2)

where α (>1) was the defector death advantage, and δ (>1) was the mutant death advantage.

To enforce a strict social dilemma, we chose parameters such that defectors had a higher birth rate than their corresponding cooperators at all resource concentrations. In other words, for *j* = 0 or 1, *b*_0*j*_(*S*) < *b*_1*j*_(*S*) for all *S*. Specifically, we set the maximum birth rate of *C* and *D* to *V*_00_ = 0.450 hr^-1^ and *V*_10_ = 0.500 hr^-1^, respectively. To reflect the relative fitness deficit of the mutants at high resource concentrations, we set the maximum birth rate of C* and D* to V_01_ = 0.293 hr^-1^ and V_11_ = 0.333 hr^-1^, respectively. A visual representation of the net growth rate (i.e., birth rate minus death rate) for each type as a function of *S* is plotted in Fig 1B.

## Birth, death, migration, mutation, and resource consumption and release

At each time step *t*, the population size of each type *N*_*ij*_(*t)* in each location was updated to reflect birth, death, migration, and mutation. We defined *b*_*ij*_(*S*), *d*_*ij*_, *m*, and *u* to be the birth rate (as a function of *S*, the instantaneous amount of resource available in that location), death rate, migration rate, and mutation rate (*u*_*D*_ or u*), respectively. We modeled the number of these events as random variables drawn from binomial distributions, each defined by *N*_*ij*_(*t)* tries and a probability of producing the respective event. Each time step interval Δ*t* was small (0.005 hrs) such that the probability of birth, death, migration, and mutation was *b*_*ij*_(*S*)Δ*t*, *d*_*ij*_Δ*t*, *m*Δ*t*, and *u*Δ*t*, respectively. To examine conditions unfavorable to cooperation, each migrant was assigned a new location at random (i.e., global migration), excluding its location of origin [51].

Because the timescale of nutrient uptake and release is much faster than cell division, we approximated *S* by calculating $\bar{S}$, the average amount of resource available over a single time step Δ*t*, using the following ordinary differential equations:

$$\begin{array}{lll} \frac{dN_{ij}}{dt} & = & N_{ij}\left[b_{ij}\left(S\right)-d_{ij}\right]\\ \frac{dS}{dt} & = & \sum_{i}\sum_{j}N_{ij}\left[\beta_{ij}\left(1-N/\kappa \right)-\gamma b_{ij}\left(S\right)\right] \end{array}$$ (3)

where *dt* ≪ Δ*t*. At each time step, *N*, *N*_*ij*_, and *S* of each location were initialized with the current values of the total population size, the population size of a given type, and the amount of resource available, respectively. All types consumed *γ* resource to produce a new cell. Resource was released by cooperators only, and *C* and C* shared the same maximum release rate *β*_0*j*_. For both types of cooperators, the resource release rate varied as a function of total population density *N* as described by *β*_0*j*_(1-*N*/*κ*), where *κ* was the carrying capacity, as has been observed with pyoverdin production in *Pseudomonas aeruginosa* [52]. Thus, release rate decreased from *β*_0*j*_ to near 0 as the population increased from a single cooperator to near the carrying capacity *κ*. The Eqs in (3) were solved numerically and *S* was integrated and averaged over Δ*t* to find $\bar{S}$. The number of births for each type was then determined by pulling from a binomial distribution *N*_*ij*_ times with probability of success $b_{ij}\left(\bar{S}\right)\Delta t$. In this way, $\bar{S}$ was deterministically calculated within each time step Δ*t*, but the number of births still stochastically fluctuated according to the binomial distribution.

**Fig 2:**
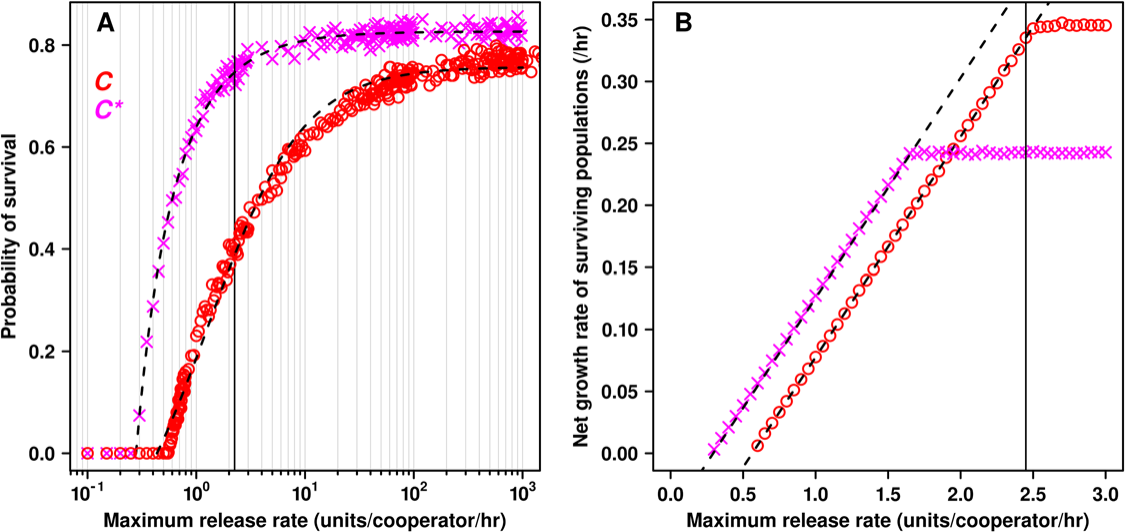
Resource release rate determines multiple aspects of fitness. Individual locations (*n* = 1024) were seeded with a single *C* (red circles) or C* (magenta X's) individual, no initial resource, no cooperator to defector mutation, and no migration. Simulations were run until every location had a population size of 0 (i.e., extinction) or ≥10^3^ individuals. The maximal release rate *β*_*0j*_ = 2.4 units/cooperator/hr used in most simulations is marked by a solid black line. **A**) The relationship between survival probability and maximum release rate *β* was fit to the equation (*p*_*max*_(*β* -*h*))/(*k+*(*β* -*h*)) using non-linear least squares regression (black dotted line). Here *p*_*max*_ was the maximum probability of survival, *h* was the smallest maximal release rate *β* allowing any surviving populations, and *k* was the *β* -*h* value allowing half-maximal probability of survival. Only rates giving survival probabilities > 0 were considered. For *C*, the parameter values were found to be *p*_*max*_ = 0.757 (0.754–0.760); *k* = 1.7 (1.6–1.8); *h* = 0.43 (0.40–0.46). For C*, *p*_*max*_ = 0.827 (0.824–0.829); *k* = 0.209 (0.201–0.217); *h* = 0.282 (0.276–0.288). We calculated the theoretical upper bound of *p*_*max*_ for *C* and found it to be <0.79 (S1 Appendix), which was in agreement with our simulations. **B**) The net growth rates of surviving populations were determined by individually fitting the growth of five populations to the equation A*exp(*gt*) using non-linear least squares regression, where *A* was the initial number of cells after population growth rate had stabilized (>tens of cells, S1 Fig), *g* was the net growth rate, and *t* was the time after the population had reached size *A*. In all cases, two standard errors were smaller than the plotting points, and were omitted. The net growth rate increased linearly as a function of maximum release rate until the amount released was greater than the amount that could be used by a population growing exponentially at its maximum net growth rate. For both *C* and C*, the slope of the linear portion (black dashed line) agreed with the theoretical value of 0.18 cooperators per unit of resource (S2 Appendix).

We chose various maximum resource release rates *β*_0*j*_. When *β*_0*j*_ = 2.4 units/cooperator/hr, a single ancestral cooperator (*C*) placed in a location without resource could grow into self-sufficient population 41% of the time (Fig 2A). As observed with invertase production in yeast, a *C* population could deterministically grow once its size had reached a critical level (S1 Fig). At a moderately large population (from tens to 10^5^ individuals, S1 Fig), *C* individuals were only slightly growth-rate limited by their own release (Fig 2B, solid black line). Using this approach allowed us to avoid the rather arbitrary assumption that more than one cooperator was required for survival [19,20,25,53]. At this release rate, a mutant C* would grow at its maximum rate which was lower than the growth rate of *C* (Fig 2B).

### Initial conditions

Each simulation tracked one metapopulation comprising 144 locations. All locations initially contained no resource. Metapopulation replicates were performed by choosing different seeds for the random number generators used in the simulation. Simulations terminated upon extinction of cooperators, or after 20,000 hrs. We determined that, in simulations where cooperators survived longer than 10,000 hours, only simulations with very low u* (<10^−10^ hr^-1^) or very high migration rates (>10^−6^ hr^-1^) occasionally resulted in cooperator extinction by 20,000 hrs. Thus, some simulations outside of this parameter range were run for 10,000 hrs.

## Results

### Defector-induced population collapse allows rescue of cooperation at intermediate migration rates

We first modeled a simple scenario unfavorable to cooperation and without C* or D* mutants. Each location of the metapopulation was occupied by a large population (10^5^ total *C* and *D* individuals) and all populations shared the same initial defector frequency of 50%. As per the Price equation or Simpson's paradox, this lack of variation in cooperator frequency among populations meant that all populations would suffer a similar fate of defector-induced population extinction. Indeed, due to the fitness advantage of *D* over *C* under all resource concentrations (Fig 1B), the frequency of *C*, as well as the amount of resource, decreased over time (S2A and S2B Fig). In most metapopulations, cooperators would go extinct before defectors in all locations, and this led to metapopulation extinction after ~200 hrs (S2C Fig). However, occasionally in one of the locations, defectors would by chance go extinct before cooperators (similar to Fig 4B). This allowed rapid growth of the resident cooperators in these defector-free locations (similar to Fig 4B), temporarily rescuing cooperation. These events would not have been observed in deterministic models, which would predict deterministic and synchronous extinction of cooperation in all locations.

**Fig 3:**
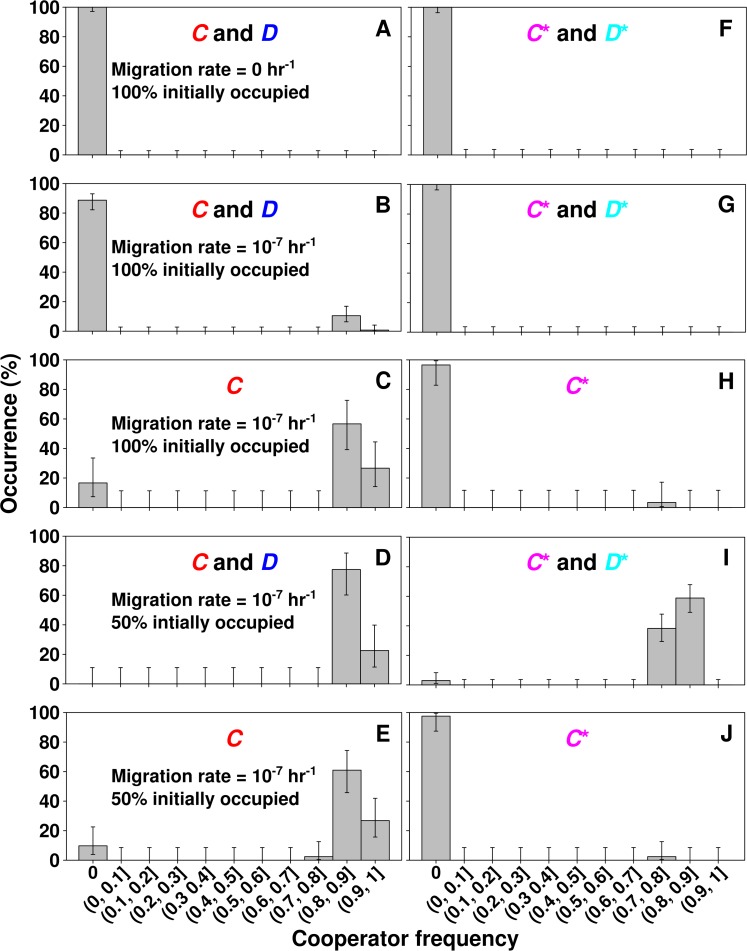
Migration, empty locations, and sometimes even the initial presence of defectors can increase the survival of cooperators. Each metapopulation was binned according to the frequency of cooperators averaged over the last 5 data points (800 hrs) of the entire simulation. **A-E**) Metapopulations with *C* and *D* only. **F-J**) Metapopulations with C* and D* only. Since the fitness of C* and D* differed from that of *C* and *D*, levels of cooperator survival differed in the two cases. **A**, **F**) In initially fully-occupied metapopulations without migration, cooperators went extinct in all metapopulations. **B**) Intermediate (10^−7^ hr^-1^) migration could promote cooperator survival frequency (15/133 in **B** compared to 0/131 in **A**; *p* < 10^−4^, Fisher's Exact Test), demonstrating that empty space arising from defector-induced extinction can sometimes rescue cooperators. **C**, **H**) Increasing the initial cooperator frequency to 100% improved cooperator survival in an initially fully-occupied metapopulation (compare to **B**, **G**). **D**, **I**) The initial presence of empty locations improved the survival of cooperation (compare to **B**, **G**). **E, J**) With initially empty locations, the initial presence of defectors can promote cooperator survival (compare **D** with **E**; **I** with **J**). Error bars indicate an estimate of the 95% confidence interval (CI) based on the Wilson method [55,56].

**Fig 4:**
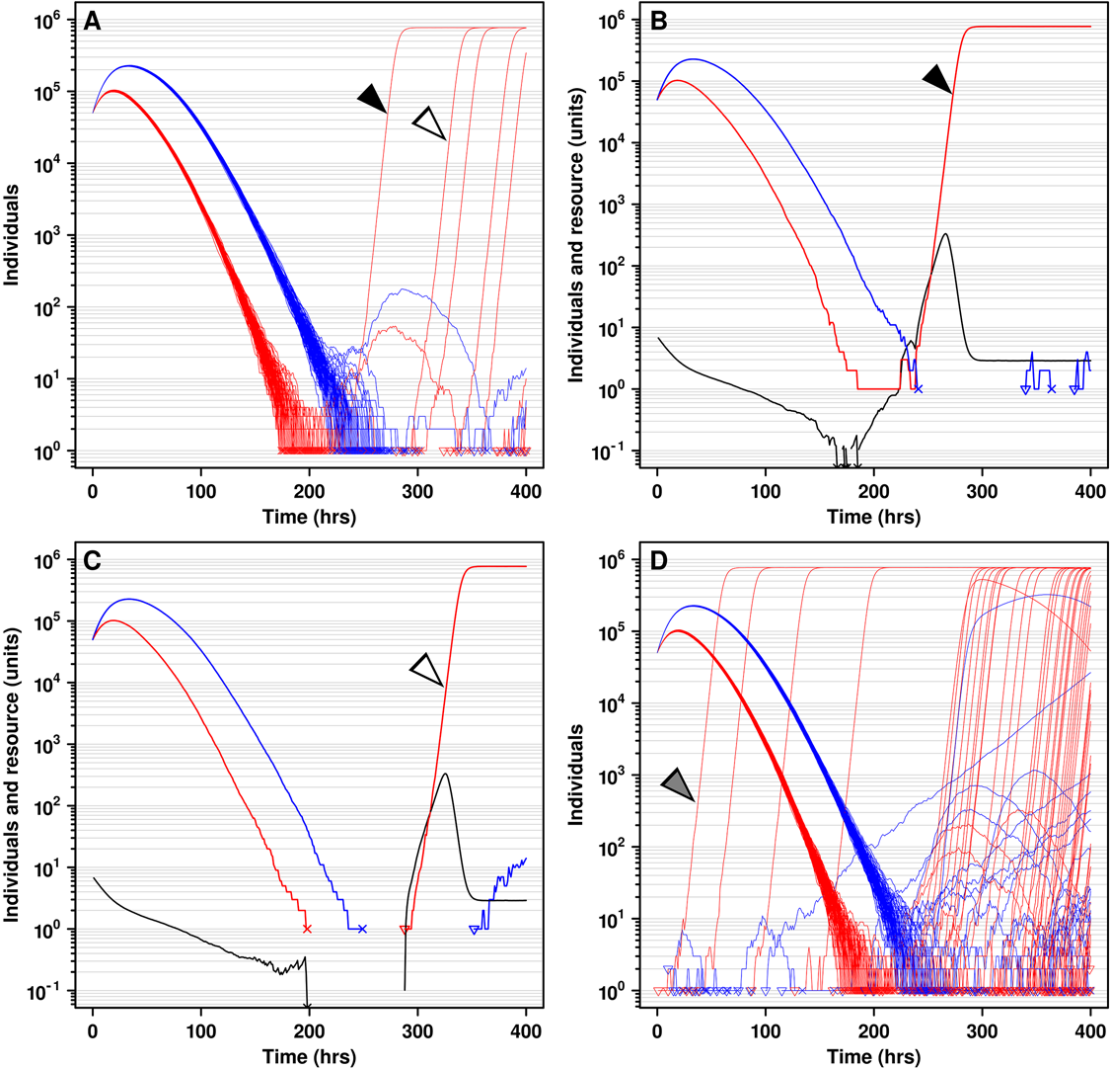
Multiple mechanisms allow cooperators to survive defector-induced population collapse. **A** and **D** each shows one example simulation of one metapopulation of *C* and *D* only, with 144 locations and a migration rate and a mutation rate *u_D_* of 10^−7^ hr^-1^. Each red or blue curve depicts the trajectory of *C* or *D*, respectively, in one location within the metapopulation. Small open triangles at the beginning of a population trajectory indicate the appearance of a new subpopulation via immigration or mutation. While we do not visually distinguish between these two types of events, one can infer, for example, that the appearance of an individual in an initially empty location must have occurred due to migration. (**A**-**C**) In an initially fully-occupied metapopulation, all empty locations must have been created by defector-induced extinction. All 144 locations of a metapopulation were initialized with 10^5^ individuals at a 1:1 *C* to *D* ratio. In an example metapopulation where cooperators achieved a high final frequency (**A**), *D* drove *C* extinct (X's at 1 on the ordinate) before suffering self-extinction in 143 out of 144 locations. However, in one location (black arrowhead, details in **B**), *D* went extinct before *C*, allowing the increase of *C* and the spread of *C* by migration to locations where *D* had driven *C* extinct (example shown by white arrowhead, details in **C**). In **B** and **C**, resource levels (black) were plotted. Downward-facing arrowhead on resource indicates that the resource concentration went below the plotted scale. (**D**) In a metapopulation with 90% locations initially occupied, *C* individuals were also able to migrate into and grow in initially empty locations (gray arrowhead).

**Fig 5:**
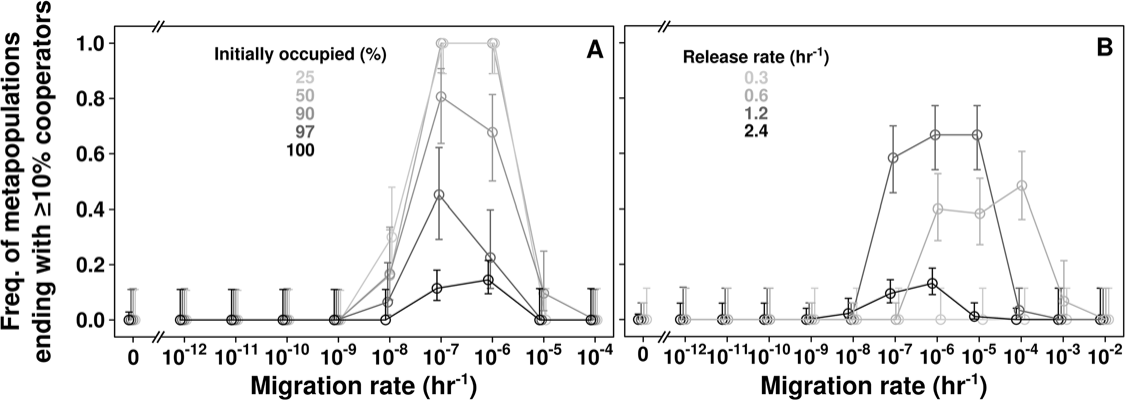
Intermediate migration rates, initially empty locations, and intermediate release rates promote the survival of cooperation. Here, we consider metapopulations of *C* and *D*. **A**) Varying the fraction of the 144 locations initially occupied by populations of 10^5^ cells at a *C* to *D* ratio of 1:1. **B**) Initially fully-occupied metapopulations as in **A**, with different cooperator release rates. Error bars were calculated as in Fig 3. In all cases, *n* ≥ 30. Note the break on the X-axis. Points are shifted slightly about the x-axis to aid visualization.

In the absence of migration, even populations of pure cooperators were short-lived: defectors would eventually rise from cooperators through mutation, and re-induce population collapse. Thus, in the absence of migration, defectors drove all metapopulations extinct (Figs 3A and 5). However, at intermediate migration rates (e.g., 10^−7^ hr^-1^, corresponding to a maximum of ~0.1 individuals migrating from each location per hr, with a generation time of ≥ 2 hrs), a fraction of initially fully-occupied metapopulations became dominated by *C* (Fig 3B and black in Fig 5A). This is because cooperators (e.g., from those locations where they happened to outlast defectors, Fig 4A and 4B) could migrate and grow in locations vacated by defector-induced extinctions (Fig 4A, 4B and 4C). Since defector migrants could not grow in empty locations, cooperators were favored over defectors in these metapopulations. Lower migration rates (<10^−9^ hr^-1^) approximated no migration, while very high migration rates (>10^−5^ hr^-1^) approximated a single, well-mixed population (Fig 5A). Thus, migration rates too low or too high were unfavorable to cooperation, as observed previously [20,33,54].

In initially fully-occupied metapopulations with an intermediate migration rate, cooperator survival improved when starting with 100% *C* (Fig 3C) instead of 50% *C* and 50% *D* (Fig 3B). Unlike the 50% *C*-50% *D* case where *D* caused population collapse with very similar dynamics in all locations (Fig 4A), in the 100% *C* case, *D* arose at somewhat different times in different locations. The latter occurred because mutation rate *u*_*D*_ was small (10^−7^ hr^-1^) compared to the local population size (~10^6^ individuals/location) (S3 Fig), and the small size of *D* meant that migration of *D* was negligible. This heterogeneity in dynamics across locations increased the chance of cooperator survival because locations vacated by early population extinctions could be colonized by surviving *C* from other locations.

The presence of initially empty locations dramatically improved cooperator survival (compare Fig 3D to 3B and Fig 5A), as also observed in other models [19,33]. In contrast to initially fully-occupied metapopulations where all empty locations used by cooperator migrants resulted from defector-induced extinction events (Fig 4A, 4B and 4C), pre-existing empty locations gave cooperators another way to escape defectors (Fig 4D). In summary, when a small number of individuals migrated, empty locations (either vacated by defector-induced population extinction or preexisting) aided the survival of cooperation. All these trends held for metapopulations comprising C* and D* only (Fig 3F, 3G, 3H and 3I), despite the fact that cooperator survival frequency differed between C*/D* metapopulations and *C/D* metapopulations.

**Fig 6:**
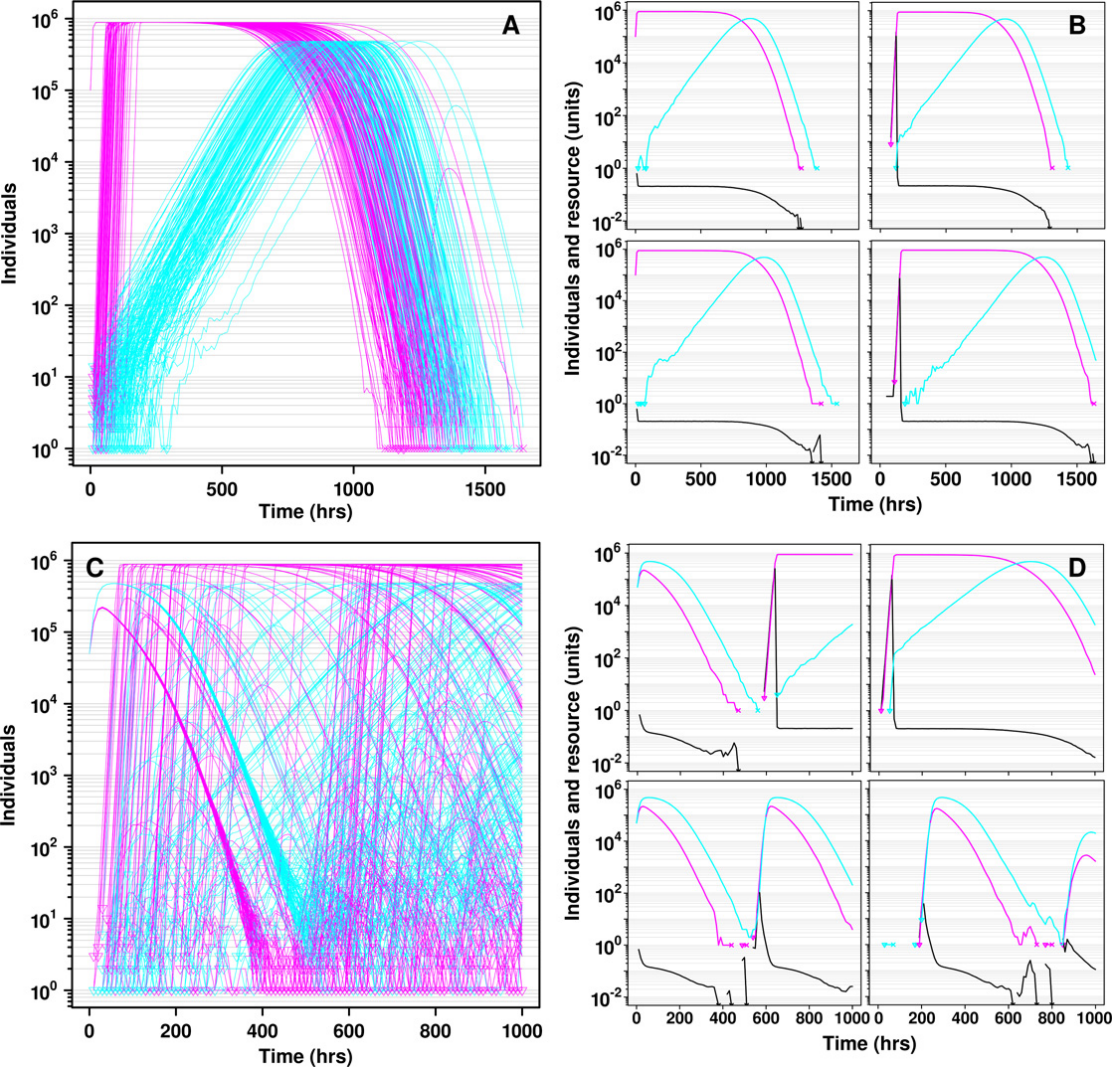
The initial presence of defectors can increase cooperator survival when empty locations are available. **A-B**) 50% of locations were initially seeded with C* cells only and they mutated to D* at *u*_*D*_ = 10^−7^ hr^-1^. **A**) All 144 locations on the same plot. **B**) Four plots of individual locations (resource in black). After the first 200 hours, initially-occupied (left) and initially unoccupied (right) locations showed the same behavior: resident or migrant C* cells grew to high density, and mutation to D* became appreciable. In all locations, although D* appeared at somewhat different times (mainly through mutation, since migration was insignificant at small population size), D* populations all slowly grew and caused local population collapse Consequently, most metapopulations went extinct. **C-D**) 50% of locations were initially seeded with C* and D* cells at 1:1. **C**) All 144 locations on the same plot. **D**) Four plots of individual locations (resource in black). While the initially-occupied locations crashed at about the same time (left), initially unoccupied locations showed different dynamics depending on whether C* or D* migrated into these locations (right). Thus, heterogeneity in dynamics promoted the survival of cooperation in these metapopulations because locations vacated by early population extinctions could be colonized by surviving C* from elsewhere.

Strikingly, at 50% initial occupancy, the initial presence of defectors increased cooperator survival (Fig 3D and 3I) compared to metapopulations starting with pure cooperators (Fig 3E and 3J). This is because, when starting with pure cooperators, cooperators in each occupied location quickly grew to saturation (Fig 6B, left), and sent migrants to other locations, which also quickly reached saturation (Fig 6B, right). In all locations, high population density led to low resource supply. Thus, defector populations, arising mainly from mutation, increased slowly (Fig 6B). This meant that cooperator populations in all locations, whether initially occupied or not, more or less synchronously reached saturation and generated slowly-rising defector populations (a band of cooperator increase, followed by a band of defector increase, followed by extinctions of cooperators and then defectors in Fig 6A). In contrast, at 50% cooperator and 50% defector, even though population dynamics initially looked homogeneous across occupied locations (Fig 6D, left), cooperator and defector migration to empty locations led to heterogeneous dynamics (Fig 6D, right). This heterogeneity across locations (Fig 6C, lack of cooperator or defector bands) favored cooperation.

All surviving metapopulations we examined displayed oscillations in cooperator frequency that dampened with time to a stable amplitude range, lasting the extent of simulations (S4 Fig). This dampening occurred as the initially chaotic population crash and stochastic rescue gave way to a more stable “chasing” dynamic (S1 Movie, S2 Movie). The fact that oscillations between cooperators and defectors were also seen in models with different assumptions and initial conditions [17,53,57–59] suggests that this type of behavior might be relatively easy to achieve.

### Intermediate cooperative benefit improves cooperator survival

The level of cooperative benefit production is a double-edged sword for the survival of cooperation. On the one hand, producing too little benefit is similar to defecting. On the other hand, excess benefit production can support defectors [26,31,46,60–64]. We found that reducing resource release rate *β* from 2.4 units hr^-1^ to 1.2 units hr^-1^ (limiting to *C*, Fig 2B) greatly improved the survival of cooperation (Fig 5B). Further reduction to 0.6 units hr^-1^ showed a less significant improvement than 1.2 units hr^-1^. This also shifted the rates of migration that supported the survival of cooperation to higher values. This is presumably because a higher migration rate would allow multiple cooperator immigrants to arrive at the same location, which can facilitate the survival of cooperators with lower release rates.

As expected, reducing the release rate to a value where a single *C* had no chance to grow into a successful colony (0.3 units hr^-1^) abolished the survival of cooperation. We used 2.4 units hr^-1^ a condition suboptimal for cooperation—in all other simulations.

### The initial presence of rare adaptive mutants, as well as moderate rates of adaptive mutantions, can promote cooperation

Resource limitation, imposed by defectors or encountered in empty or densely-populated locations, can select for mutants adapted to resource limitation. Because of the fitness trade-off (Fig 1B), these mutants will be kept at low frequencies when resource is abundant (S5 Fig). We reasoned that the initial rarity of these mutants could help rescue cooperation from defectors, similar to the effect of small population size caused by defector-induced population collapse or individual migration into empty locations.

**Fig 7:**
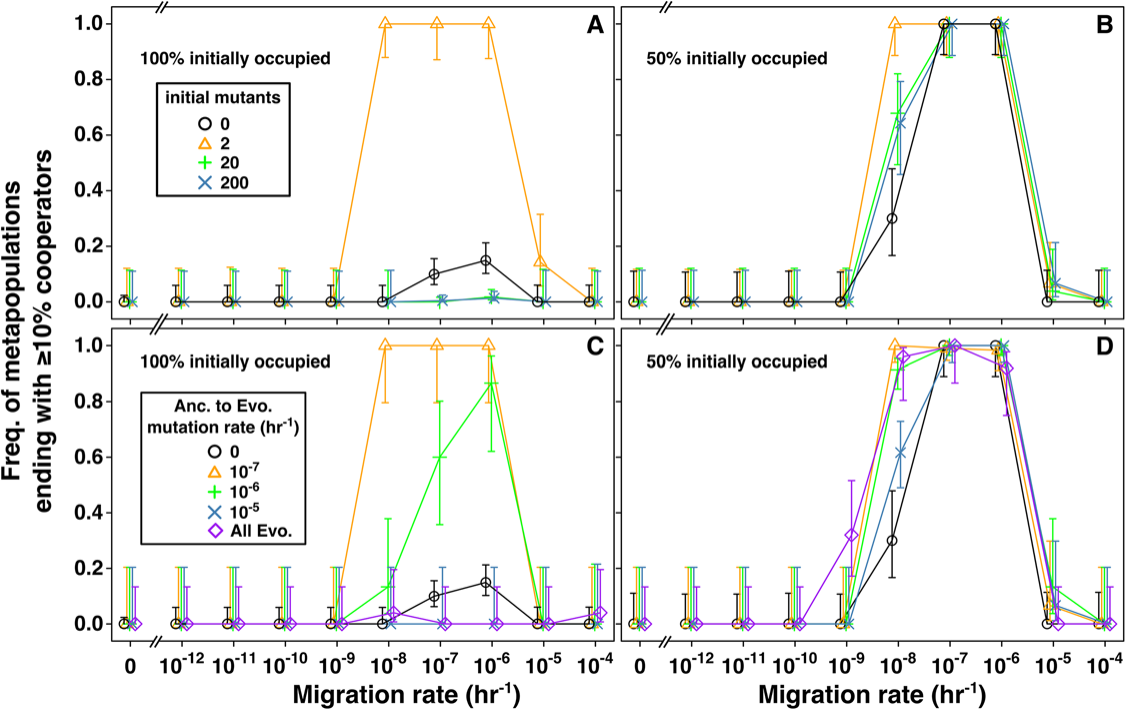
High-fitness mutants can improve the survival of cooperation. **A-B**) Non-empty locations in each simulation were initialized with a total of 10^5^ cells at a cooperator to defector ratio of 1:1. Each of these populations were seeded with a total of 0 (black circle), 2 (orange triangle), 20 (green plus), or 200 (blue x) initial mutants, also at a C* to D* ratio of 1:1. **A**) 100% of locations initially occupied. **B**) 50% of locations initially occupied. **C-D**) Instead of seeding the initial mutants, initial *C* and *D* populations were given an ancestor to evolved mutation rate u* of 0 (black circle), 10^−7^ (orange triangle), 10^−6^ (green plus), or 10^−5^ (blue x) hr^-1^. At an extremely high mutation rate, cooperator survival should approximate that in C*/D* metapopulations, plotted here for comparison (purple diamond). Trends in **C** and **D** are qualitatively similar to those in **A** and **B**. Data is slightly shifted about the x-axis to aid visualization of overlapping points.

To gain intuition about how adaptive mutants might help rescue cooperation from defectors, we initiated metapopulations with a mixture of *C* and *D* (initially 100% occupied, 10^5^ total cells/population, 1:1 *C*:*D*) so that defectors could induce resource limitation. When we added a small number (Fig 7A, orange triangles) but not a large number (green pluses and blue crosses) of C* and D* to every population, cooperator survival improved compared to the no-mutant scenarios (black circles). This appeared to be general, holding regardless of initial occupancy (Fig 7B) or details of the fitness relationships between the four types (S6A and S6B Fig). The only exception was that rare initial mutants might not improve cooperation when cooperator survival was already high (S6C Fig).

**Fig 8:**
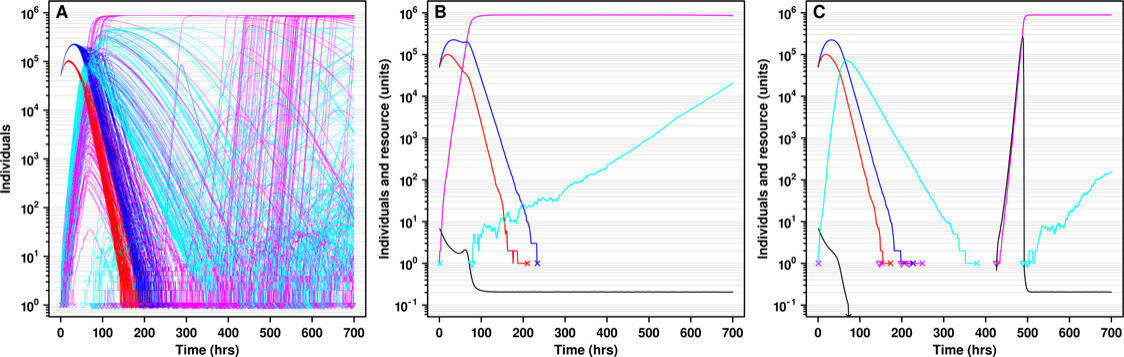
Under defector-induced resource limitation, mutant dynamics dominate metapopulation dynamics. **A**) An example metapopulation initialized with 100% of its locations occupied. Each population initially contained 1 C* and 1 D*. Each curve shows the population size of *C* (red), *D* (blue), C* (magenta), or D* (cyan) in one location of a metapopulation. In most locations, D* drove both C* and itself extinct. **B**) In some locations, C* initially outlived D* by chance. C* then rose to high frequency and outcompeted both *C* and *D*. New D* were generated by mutation of C*. **C**) C* individuals from populations depicted in **B** could migrate into other locations that were emptied due to extinction induced by D*.

Rare initial mutants promoted cooperation because, under resource limitation precipitated by defectors, the population dynamics of a metapopulation were dominated by the dynamics of mutants due to their high resource affinity (low *K*) compared to ancestors (Fig 8). When the initial C* and D* were rare but not when they were abundant, it was almost guaranteed that in at least one population, D* would go extinct before C* by chance. The remaining C* would then rapidly increase into a large population (Fig 8B) and, at intermediate migration rates, spread to locations emptied by D*-induced extinction (Fig 8C). In these metapopulations, oscillations between C* and D* were also observed (S4C and S4D Fig).

To test whether continuous mutation from *C* and *D* to C* and D* could support cooperator survival, we varied u* in metapopulations initiated with 1:1 *C*:*D*. As expected, at very high mutation rates (e.g., Fig 7C and 7D, blue), cooperator survival frequency resembled that of metapopulations initiated with C* and D*, which in turn differed greatly depending on the initial occupancy of metapopulation (Fig 7C and 7D, purple). Otherwise, except when u* was unrealistically low (S7A and S7B Fig, orange) or zero (Fig 7C and 7D, black), cooperator survival appeared to be largely independent of the initial occupancy of the metapopulation (S7A and S7B Fig, blue and green; Fig 7C and 7D, orange and green). Overall, unless cooperator survival was already very high without mutants (S7B Fig), moderate u* improved cooperator survival (Fig 7C and 7D, orange and green; S7A Fig, grey, blue and green; all compared to black).

### The initial presence of defectors allows adaptive mutants to promote cooperation

**Fig 9:**
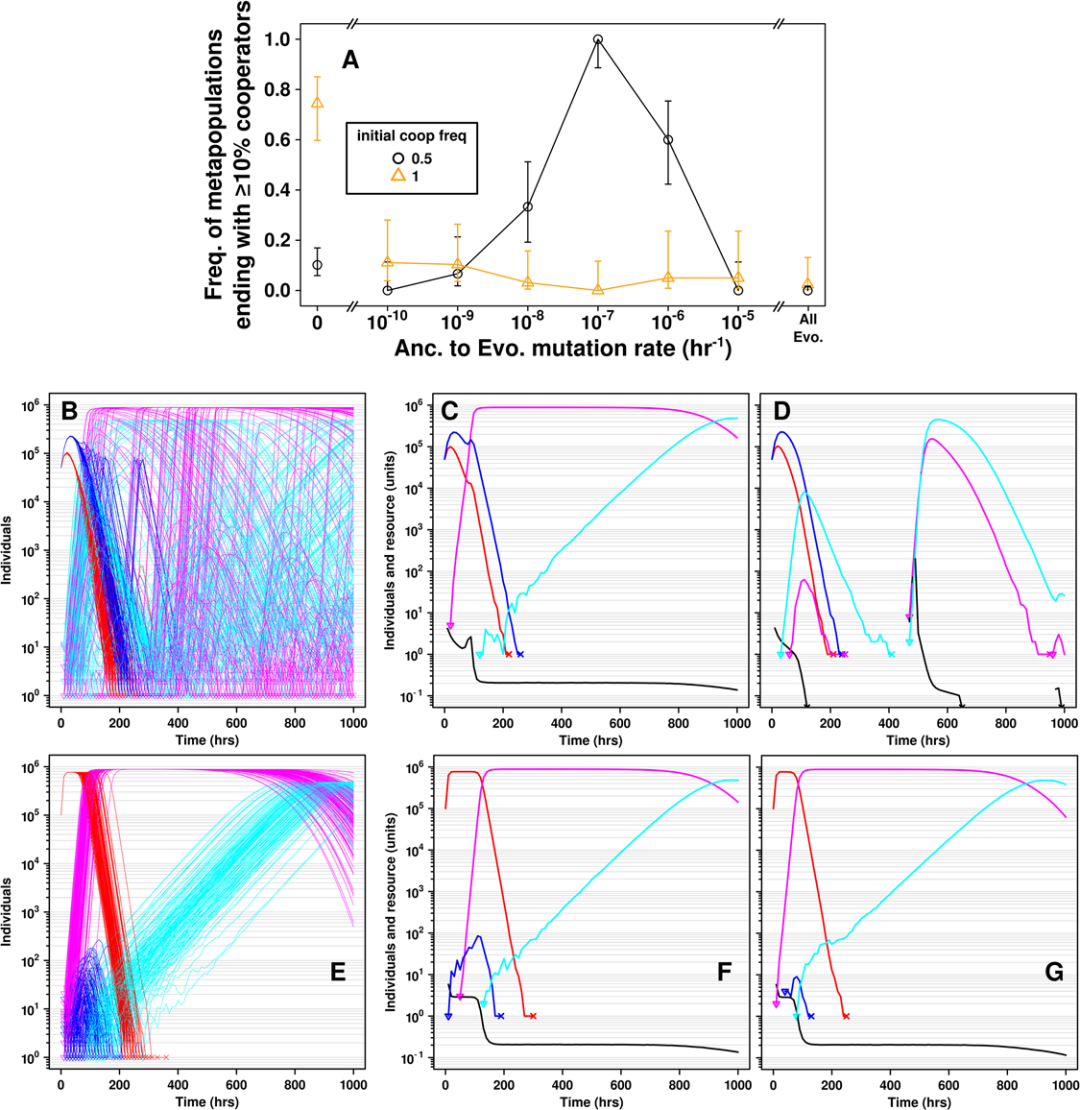
The initial presence of defectors allows moderate mutation rates to improve cooperator survival. **A**) Simulations were initialized with 10^5^ cells in all locations. The migration rate *m* and the mutation rate *u*_*D*_ were 10^−7^ hr^-1^. Initial cooperator frequency was 50% (black circle) or 100% (orange triangle). **B-D**) One simulation initiated with 50% cooperators. The initial presence of defectors increased heterogeneity in dynamics due to the different timing of appearance of C* and D* in different locations. This heterogeneity allowed cooperation to persist over time. **E-G**) One simulation with 100% cooperators. In all locations, C* quickly outcompeted *C* and *D* and rose to high density. Mutation from C* to D* formed a “band” of D* growth across all locations (migration of D* was initially negligible due to the relatively small number of D* in each location). This resulted in a poor outcome for cooperation, since all populations eventually collapsed at about the same time, limiting the opportunity for cooperators to escape to empty locations. 50% initially-occupied metapopulations showed a similar phenomenon.

The initial presence of defectors allowed moderate u* to promote cooperator survival (Fig 9, black). In contrast, metapopulations initiated with 100% *C* did not benefit from moderate u* (Fig 9, orange). This is because in the absence of initial *D*, *C* mutated to C* which quickly migrated and dominated all locations (Fig 9E, 9F and 9G). This in turn triggered a synchronous rise of D* in all locations (Fig 9E, 9F and 9G), which was detrimental to cooperator survival compared to when defectors were initially present (where D* could contribute to de-synchronization, Fig 9B, 9C and 9D). Thus, the initial presence of *D* in conjunction with moderate u* can promote the survival of cooperation.

In natural populations, we expect defectors to be present due to mutation from cooperators. We also expect that there will be many types of mutants, each conferring a different fitness improvement over the ancestor in resource-limited environments. The fittest C* and D* that survive genetic drift will dominate a metapopulation. Regardless of the average fitness of the population, the fittest mutants are expected to be relatively rare (by definition), but exist (since being the fittest is relative to other mutants) [65–67]. Thus, this mechanism of reducing population size could promote cooperation even as the population evolves.

## Discussion

Defectors, by degrading the environment through resource exploitation, create small population sizes that can help rescue cooperation from defectors. This happens via three mechanisms. First, through resource depletion, defectors directly reduce population size and, occasionally, cooperators outlive defectors by chance (Fig 4A and 4B). Second, while pre-existing empty locations aid the survival of cooperation (Figs 3 and 5A, [19,33]), locations emptied by defector-induced extinction can also promote the survival of cooperation by supporting the growth of migrant cooperators but not defectors (Figs 4C and 8C). In fact, the initial presence of defectors often increases cooperator survival (Figs 3, 6 and 9). This is because, relative to metapopulations starting with pure cooperators, defectors can increase the heterogeneity in population collapse dynamics, reducing the chances that all populations go extinct simultaneously. Third, selection for rare mutants adapted to resource limitation, which can be induced by defectors, introduces another population bottleneck (Fig 8). This facilitates the survival of cooperation (Fig 7) when defectors are initially present (Fig 9). Thus, in structured populations with intermediate migration rates, defectors themselves can help rescue cooperation. These relatively rare events (e.g., mutation, migration, chance survival of cooperators) captured by our stochastic, agent-based modeling approach may be an important contributor to the success of cooperation over evolutionary time.

Defector-induced local population extinction has been observed in microbes. For example, when the social bacterium *Myxococcus xanthus* encounters starvation, individuals aggregate into fruiting bodies. In a fruiting body, a small fraction of cells become stress-resistant spores while the rest undergo autolysis or form the peripheral shell of the fruiting body. When mixed with cooperators, obligate defectors incapable of developing into fruiting bodies on their own can exploit cooperators and have a disproportionately large contribution to spore formation. Some of these obligate defector types can drive the entire mixed population to extinction during cycles of famine and feast [68]. A collapsing population can be rescued when an obligate defector mutates into a super-cooperator [69].

We have observed that too much or too little resource production is detrimental to cooperator survival (Fig 5B). Experimentally, siderophore-producing bacteria readily evolve to reduce siderophore production if exposed to extremely iron-limited conditions, but complete loss of siderophore production was rarely observed [46]. The authors of that study considered reduction in resource production a type of defecting. However, if cooperation is defined as the production of a common good—and not the amount of common good produced—our results (Fig 5B) suggest that evolution to a lower resource release rate might be an effective way for cooperators to survive non-producing defectors. Intriguingly, they also found that reduced investment in siderophore production was not associated with reduced population growth rates, suggesting that the evolved populations may have also adapted to their nutrient-limited environment.

Populations with high defector load show decreased growth rates and eventually go extinct because defectors impose severe resource limitation. In microbial populations, resource limitation will rapidly select for mutants adapted to this new environment [42,44,70]. Despite this fact, most models have only considered migration rate [53] and investment in resource production [20,25,71] as evolvable parameters. One group modeled what would happen if cooperators and defectors had to adapt to an evolutionary pressure unrelated to cooperation, such as a predator or parasite [72]. They found positive frequency-dependent effects: if cooperators were initially dominant, it was harder for defectors to invade, and vice versa. They speculated that this occurred because the numerically dominant population was more likely to attain a better fitness-enhancing mutation than the minority population [72].

We found that externally-generated selective pressures were not necessary to help the survival of cooperation. Instead, the rise of defectors could create an environment favoring mutants adapted to defector-induced resource limitation. Mutants adapted to resource limitation probably exist in any microbial population of sufficient size [42,65–67]. We did not model multiple types of mutants with fitness differences because mutants with smaller fitness advantages would be displaced by mutants with larger fitness advantages. We propose that only the population size of the “best” type of mutant will determine the survival of cooperation. We therefore predict that the population size of the mutant conferring the largest fitness advantage will determine the overall frequency of the survival of cooperation. If these mutants pre-exist at a low level (Fig 7A and 7B), or occur at a moderate rate (Fig 7C and 7D and S7A Fig), they can promote cooperator survival.

The mutation scenarios assumed in our models are likely to occur. Theoretical [65] work and experiments with bacteria [66] and viruses [73] suggest that the fitness distribution of adaptive mutations may be exponentially distributed and invariant to the mean fitness of the population regardless of biological details. Recent work suggests that the fitness distribution of adaptive mutations is exponential for modest fitness gains, but exhibits a flat tail for highly adaptive mutants [67]. Thus, high-fitness mutants are likely to pre-exist [48] regardless of the mean fitness of the population, but at low levels due to their rarity and the high probability that they will be less fit than the ancestor in the ancestral environment. The large range (several orders of magnitude) of moderate u* capable of promoting cooperation is also likely to be met in reality. Thus, in sufficiently large populations, selection for high-fitness mutants could in principle help the survival of cooperation through repeated rounds of defector-induced collapses, even as the population evolves improved starvation tolerance.

We join others in advocating for modeling techniques that narrow the gap between theory and experiment by basing parameters on physically measurable quantities [36,74]. Our model assumptions were based on measurable physical parameters, such as birth, death, and release and consumption rates. While we chose specific parameters to model mutants with fitness trade-offs, our modeling framework is general, and is designed to handle arbitrary numbers of types with arbitrary fitness functions, and can therefore be used to model many types of ecological interactions.

## Methods

### Implementation

The modeling framework was written in Java and is available on GitHub at https://github.com/nodice73/metapop. All data was analyzed with code written in the R programming language [75] and is available in the file 'metapop.r', which is included in the source tree for the simulation framework.

### Re-running simulations

Each simulation was assigned a “universally unique identifier” (UUID) by placing the output of the simulation into a folder named with this UUID. A separate file produced by the script running the simulation recorded the arguments used to run the simulation. In addition to the parameters used for the simulation, these arguments also included the set of random numbers used to seed the appropriate random number generators, as well as the location of the simulation (which included the UUID). The UUID of a simulation was used to look up the set of seeds which initialized its RNGs (Random Number Generators). These seeds were used to exactly replicate the output of the original run while, for example, reducing the amount of time it was run or increasing the frequency of output data. These re-runs also received their own UUIDs.

## Supporting Information

S1 Fig Dynamics of populations initiated with a single ancestral cooperator. Twenty randomly-chosen populations from the *β* = 2.4 units/cooperator/hr release rate described in Fig 2. Extinction is indicated by an 'X' at 10^0^. Even though 10^6^ was the carrying capacity, the maximum steady state population size was below 10^6^ because at that population size, birth due to reduced resource release balanced death.
(TIFF)

S2 Fig Dynamics of defector-induced population collapse. A metapopulation without migration initialized with 10^5^ individuals at a 1:1 *C* to *D* ratio in 100% of the 144 locations experienced complete destruction of cooperation. **A**) Metapopulation-wide cooperator frequency decreased over time. **B**) A single location in the metapopulation showing the number of *C* (red), *D* (blue) individuals and the amount of resource (black). Black arrowhead shows the resource level dropping below the plotted resource range. **C**) Dynamics of *C* and *D* in all locations of the metapopulation.
(TIFF)

S3 Fig Dynamics of simulations started with 100% *C*. Metapopulations were initialized with all 144 locations initially occupied with *C* only and a migration rate of 10^−7^ hr^-1^. The initial metapopulation-wide population collapse could result in the survival (**A**) or extinction (**B**) of cooperation. In both **A** and **B**, *D* appeared in and caused local population collapse at more heterogeneous times than if all locations in a metapopulation were initiated with 50% *C*-50% *D* (compare with Fig 4A).
(TIFF)

S4 Fig Examples of dampened oscillations in metapopulation-wide cooperator and defector population sizes and frequencies. **A**) The total population size of *C* (red) and *D* (blue) in metapopulations initialized with 100% initial occupancy and a migration rate of 10^−7^ hr^-1^. **B**) The frequency of cooperators in **A**. **C**) The total number of *C* (red), *D* (blue), C* (magenta), and D* (cyan) in metapopulations initialized as in (**A**), except 1 C* and 1 D* individual were added to each population at the beginning of the simulation. **D**) The frequency of cooperators in (**C**).
(TIFF)

S5 Fig *C* has an advantage over C* in the absence of defectors before population saturation. 1024 locations initialized with 49,750 *C* and 250 C* were run without migration. The average ratio of C to C* over the first 4 hours (before saturation was reached) increased (green line). The average growth rate was 0.2895 (0.2893–0.2897) hr^-1^ for *C* and 0.2381 (0.237–0.239) hr^-1^ for C*. Note the scale of the y-axis is logarithmic.
(TIFF)

S6 Fig A small number of initial mutants adapted to resource limitation can help cooperation even when D* had an even higher fitness than that in Fig 1 (Table 1). **A**) Note that the crossing point where the fitness of C* became less than *C* occurred before the crossing point where the fitness of D* became less than the fitness of *D*. In Fig 1, this relationship was reversed. Results for 100% (**B**) and 50% (**C**) initially occupied metapopulations are shown. Simulation conditions were otherwise identical to those shown in Fig 7A and 7B. Since the parameters for *C* and *D* were unchanged, the data for 0 initial mutants is the same as Fig 7A and 7B.
(TIFF)

S7 Fig Compared to moderate mutation rates, extremely low mutation rates can be detrimental to cooperation. Simulations were initialized with either 100% (**A**) or 50% (**B**) of the locations initially occupied. Non-empty locations in each simulation were initialized with a total of 10^5^ cells and a *C* to *D* ratio of 1:1. The mutation rate from ancestor to evolved (u*) was varied from 0 (black circle), 10^−10^ (orange triangle), 10^−9^ (green plus), 10^−8^ (blue x), or 10^−7^ (grey triangle) hr^-1^. Simulations initiated with 100% evolved (purple diamond) are included for comparison. Data is slightly shifted about the x-axis to aid in visualization of overlapping data points. **C**-**H**) Simulation dynamics with 50% of locations initially occupied, a migration rate of 10^−7^ hr^-1^, and u* of 10^−9^ hr^-1^ (**C-E**) or 10^−7^ hr^-1^ (**F**-**H**). **C**-**E**) An extremely low mutation rate can hinder the survival of cooperation. **C**) All 144 locations of a single simulation that eventually went extinct. **D-E**) Since u* (10^−9^ hr^-1^) was very low, ancestor to evolved mutation occurred rarely. At a migration rate of 10^−7^ hr^-1^, *C* dominated the metapopulation (**B**, black). Thus, initially, *C* to C* mutation was much more likely to occur than *D* to D* mutation. Once a C* mutant appeared, it would quickly rise to high frequency (black arrowhead in **C** and **D**). Since the migration rate (10^−7^ hr^-1^) was much greater than u*, the initial C* population quickly spread throughout the metapopulation (one example is indicated by the white arrowhead in **C** and **E**). New migrant C* arrived around the time ancestral types were near carrying capacity, were collapsing, or had gone extinct, which meant that resource was low (e.g., **E**). This allowed C* to outcompete the remaining ancestor types throughout the metapopulation. However, this coordinated sweep of C* caused a “band” of D* to appear due to mutation. Thus, all locations became synchronized, causing subsequent defector-induced population collapses to occur at similar times, which decreased the chances of cooperator survival compared to moderate u*. (**F**-**H**) At a moderate u* (10^−7^ hr^-1^), increased heterogeneity in the timing of mutant appearance improved the survival of cooperation compared to very low u*. Dynamics of the entire metapopulation (**F**) lacked “bands” of C* and D*. Indeed, individual locations (examples in **G** and **H**) showed heterogeneous dynamics in C* and D*. The broader span of the appearance times of evolved mutants resulted in “chasing” dynamics in the metapopulation that proved beneficial to the survival of cooperation.
(TIFF)

S1 Appendix Calculation of the maximum probability of survival of a single cell. (PDF)

S2 Appendix Calculation of the expected slope for growth rate versus release rate. (PDF)

S1 Movie Oscillations in an initially 100% occupied metapopulation. The metapopulation was initialized with 100% of its locations occupied with 10^5^ individuals at a ratio of 1:1 *C*:*D*. *u*_*D*_ = 10^−7^ hr^-1^, *m* = 10^−7^ hr^-1^. Color hue indicates frequency of cooperators, going from red (100% cooperator) to blue (100% defector) through purple (50% cooperator, 50% defector). Color opacity indicates population density.
(MP4)

S2 Movie Oscillations in an initially 50% occupied metapopulation. Same as S1 Movie, only the metapopulation was initialized with 50% of its locations initially occupied.
(MP4)

S1 Data Data from simulations without ancestor to evolved mutation. (CSV)

S2 Data Data from simulations with ancestor to evolved mutation. (CSV)

S3 Data A zip file containing data used to calculate survival probability and growth rates (Fig 2). (ZIP)
