# Supplementary figures and images for: Defectors Can Create Conditions That Rescue Cooperation
Source: PLoS Comput Biol. 2015 Dec 21;11(12):e1004645. doi: 10.1371/journal.pcbi.1004645 (PMC4687000; doi:10.1371/journal.pcbi.1004645)

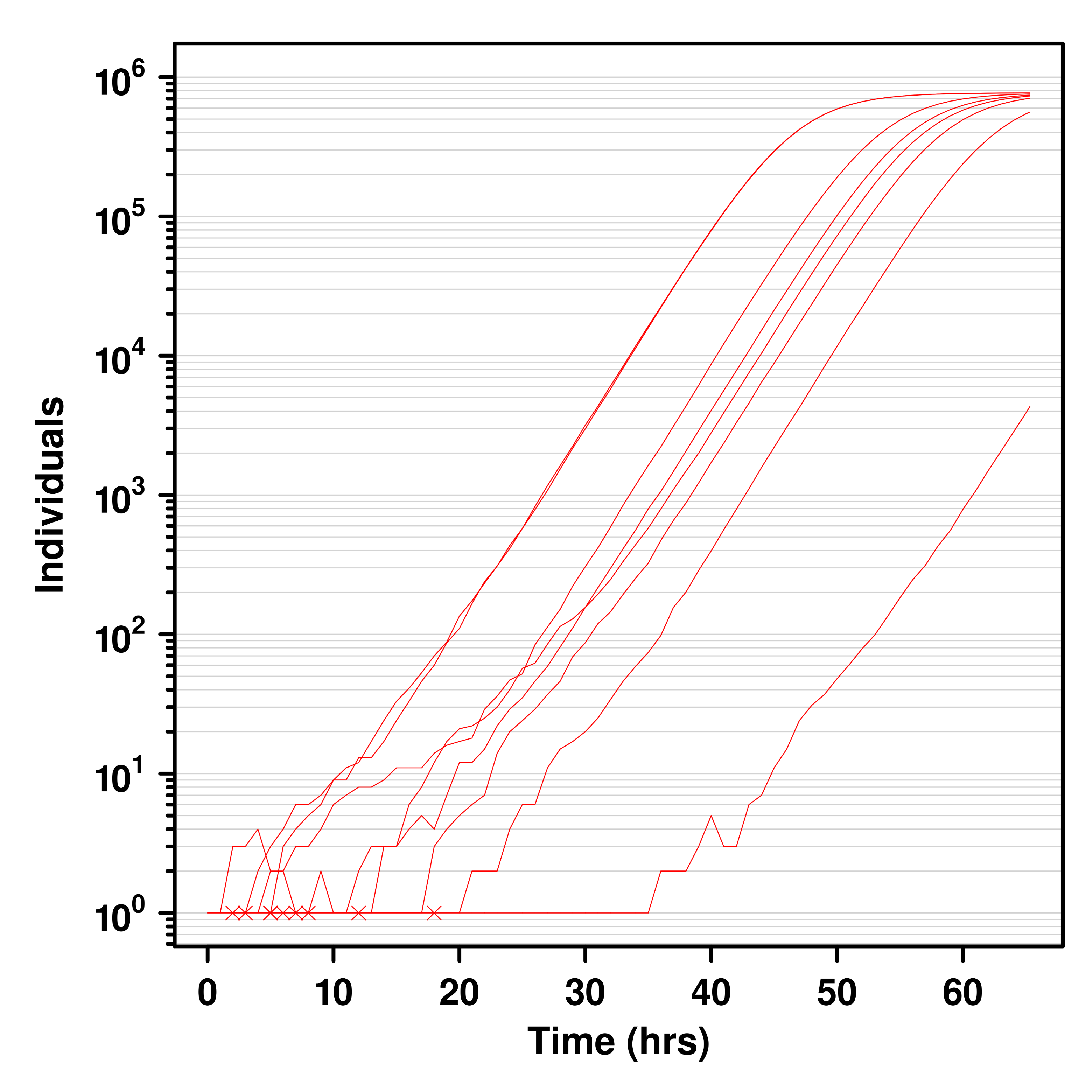

Supplement: S1 Fig — Twenty randomly-chosen populations from the β = 2.4 units/cooperator/hr release rate described in Fig 2. Extinction is indicated by an 'X' at 100. Even though 106 was the carrying capacity, the maximum steady state population size was below 106 because at that population size, birth due to reduced resource release balanced death. (TIFF) [file pcbi.1004645.s001.tiff]

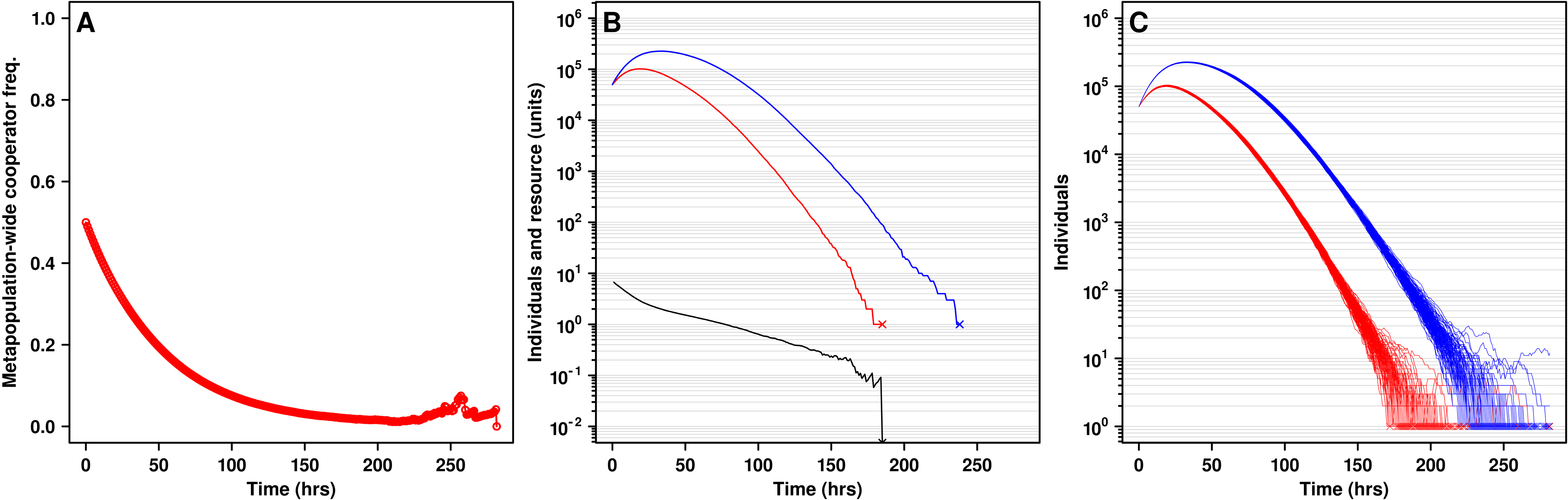

Supplement: S2 Fig — A metapopulation without migration initialized with 105 individuals at a 1:1 C to D ratio in 100% of the 144 locations experienced complete destruction of cooperation. A) Metapopulation-wide cooperator frequency decreased over time. B) A single location in the metapopulation showing the number of C (red), D (blue) individuals and the amount of resource (black). Black arrowhead shows the resource level dropping below the plotted resource range. C) Dynamics of C and D in all locations of the metapopulation. (TIFF) [file pcbi.1004645.s002.tiff]

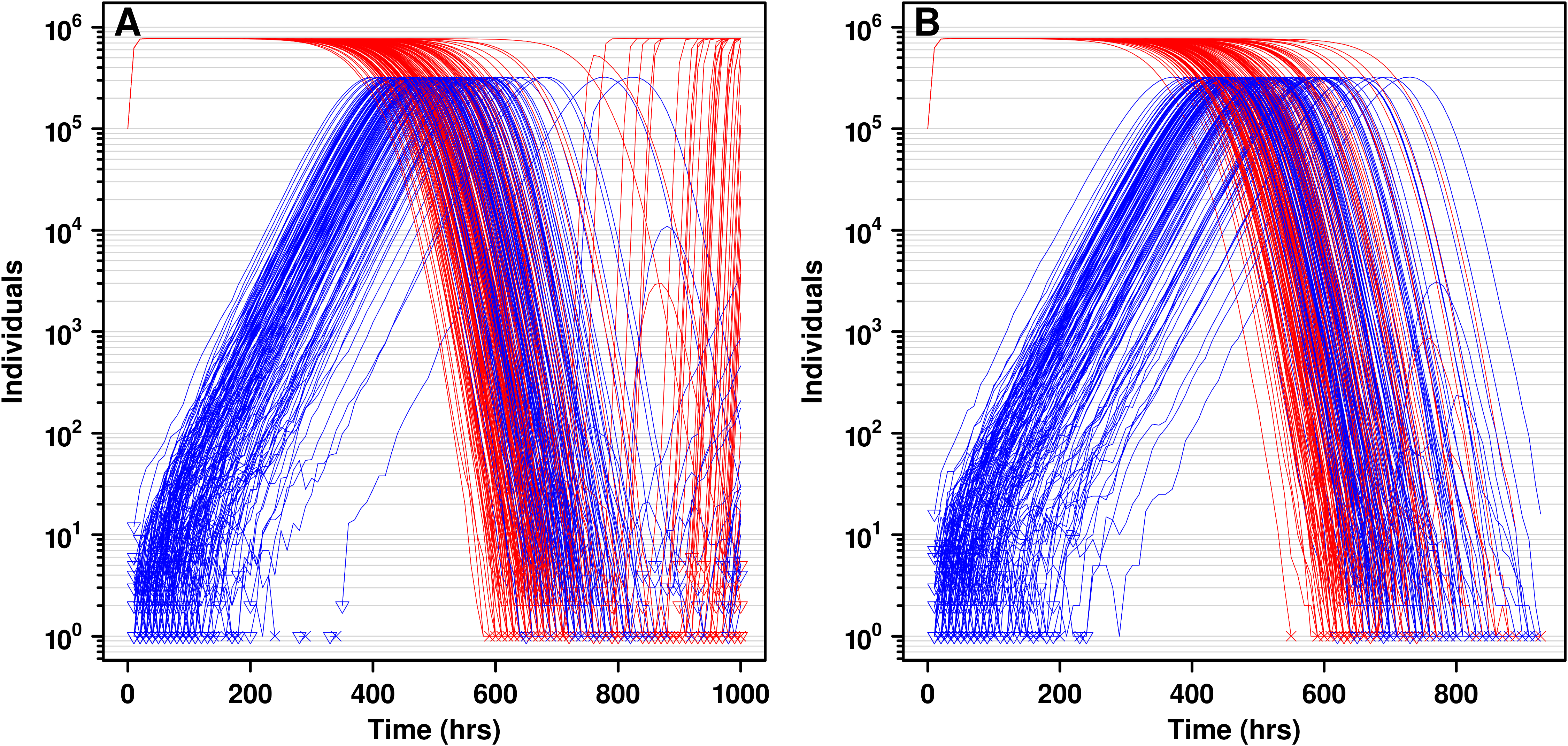

Supplement: S3 Fig — Metapopulations were initialized with all 144 locations initially occupied with C only and a migration rate of 10−7 hr-1. The initial metapopulation-wide population collapse could result in the survival (A) or extinction (B) of cooperation. In both A and B, D appeared in and caused local population collapse at more heterogeneous times than if all locations in a metapopulation were initiated with 50% C-50% D (compare with Fig 4A). (TIFF) [file pcbi.1004645.s003.tiff]

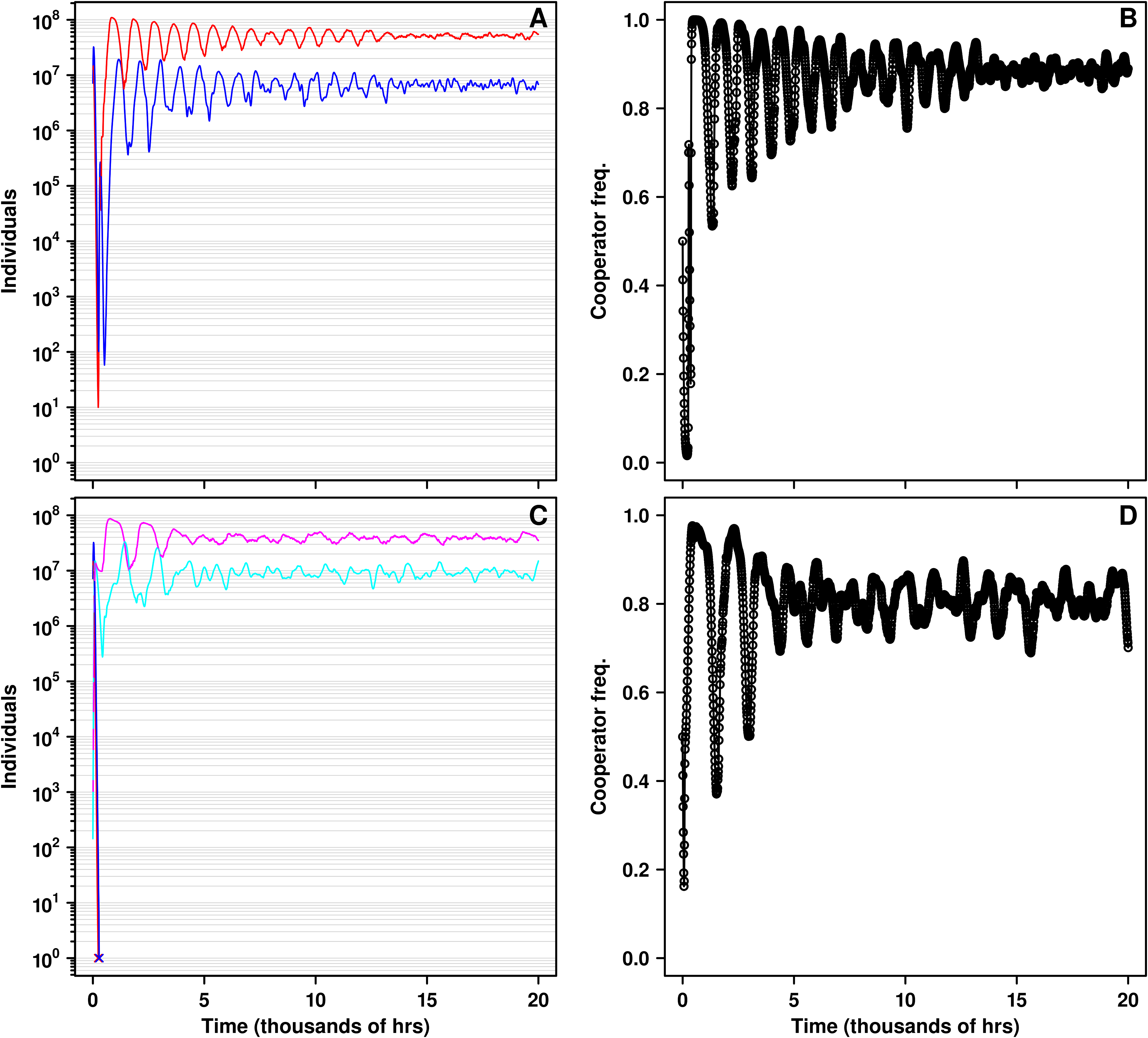

Supplement: S4 Fig — A) The total population size of C (red) and D (blue) in metapopulations initialized with 100% initial occupancy and a migration rate of 10−7 hr-1. B) The frequency of cooperators in A. C) The total number of C (red), D (blue), C* (magenta), and D* (cyan) in metapopulations initialized as in (A), except 1 C* and 1 D* individual were added to each population at the beginning of the simulation. D) The frequency of cooperators in (C). (TIFF) [file pcbi.1004645.s004.tiff]

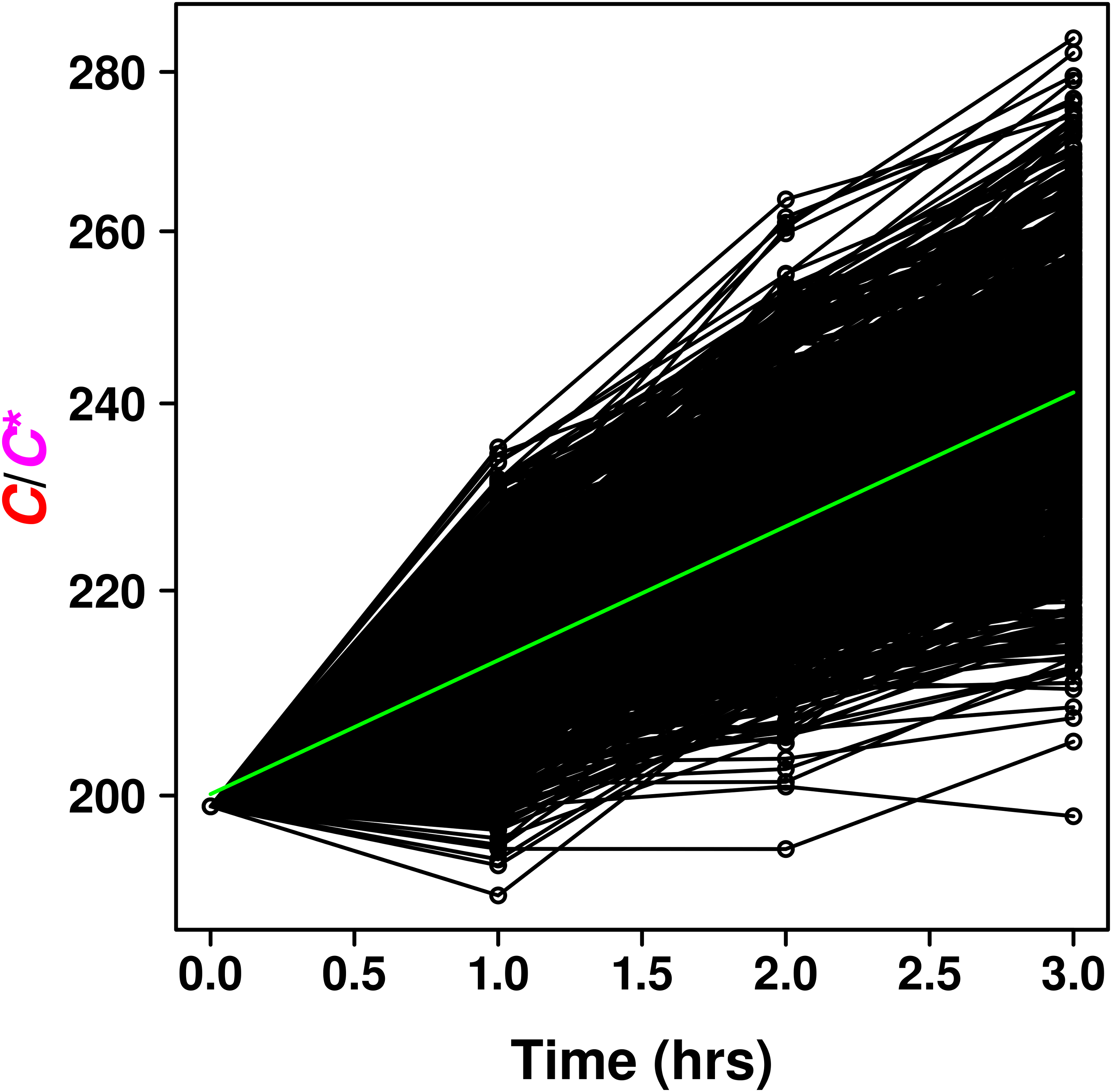

Supplement: S5 Fig — 1024 locations initialized with 49,750 C and 250 C* were run without migration. The average ratio of C to C* over the first 4 hours (before saturation was reached) increased (green line). The average growth rate was 0.2895 (0.2893–0.2897) hr-1 for C and 0.2381 (0.237–0.239) hr-1 for C*. Note the scale of the y-axis is logarithmic. (TIFF) [file pcbi.1004645.s005.tiff]

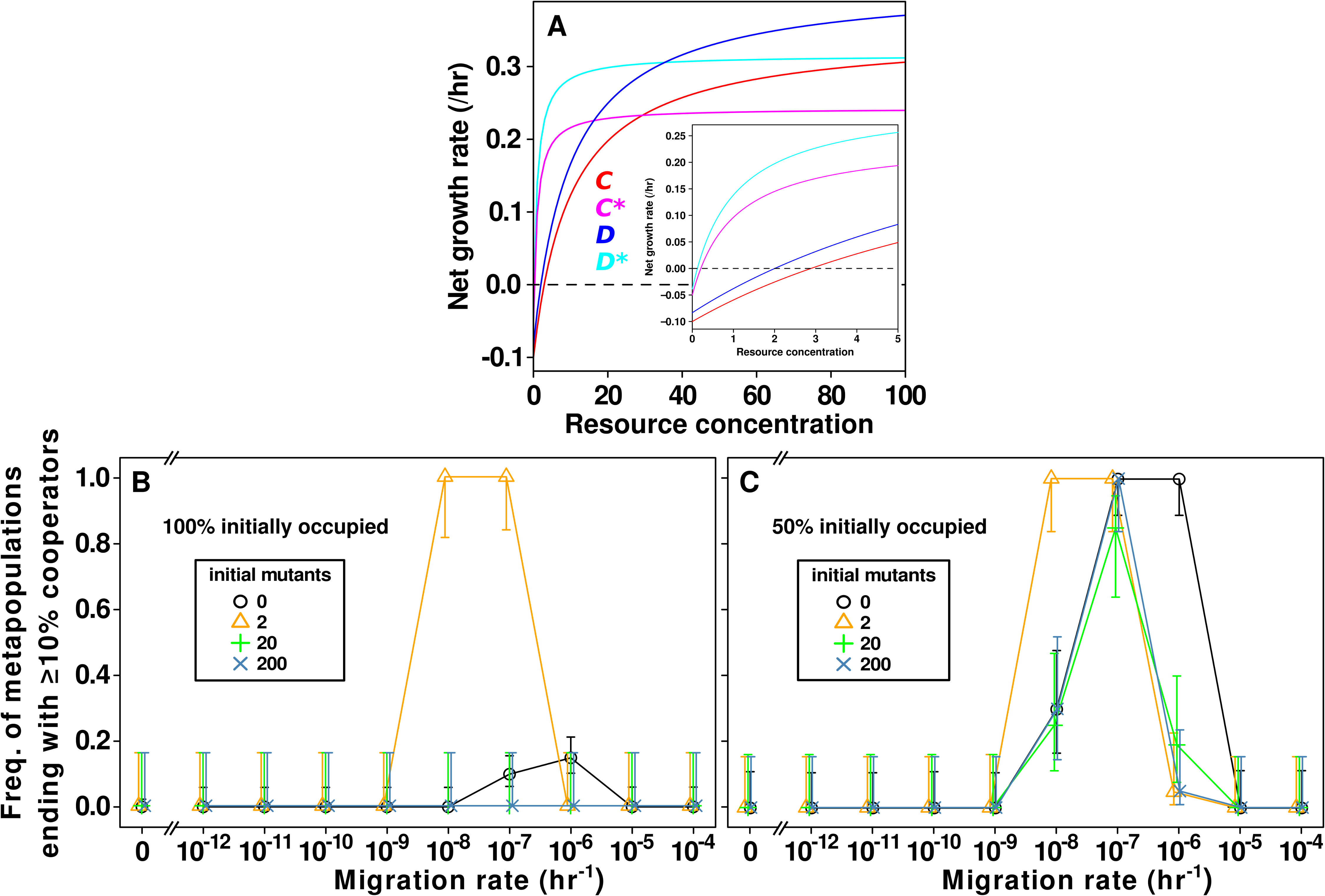

Supplement: S6 Fig — A) Note that the crossing point where the fitness of C* became less than C occurred before the crossing point where the fitness of D* became less than the fitness of D. In Fig 1, this relationship was reversed. Results for 100% (B) and 50% (C) initially occupied metapopulations are shown. Simulation conditions were otherwise identical to those shown in Fig 7A and 7B. Since the parameters for C and D were unchanged, the data for 0 initial mutants is the same as Fig 7A and 7B. (TIFF) [file pcbi.1004645.s006.tiff]

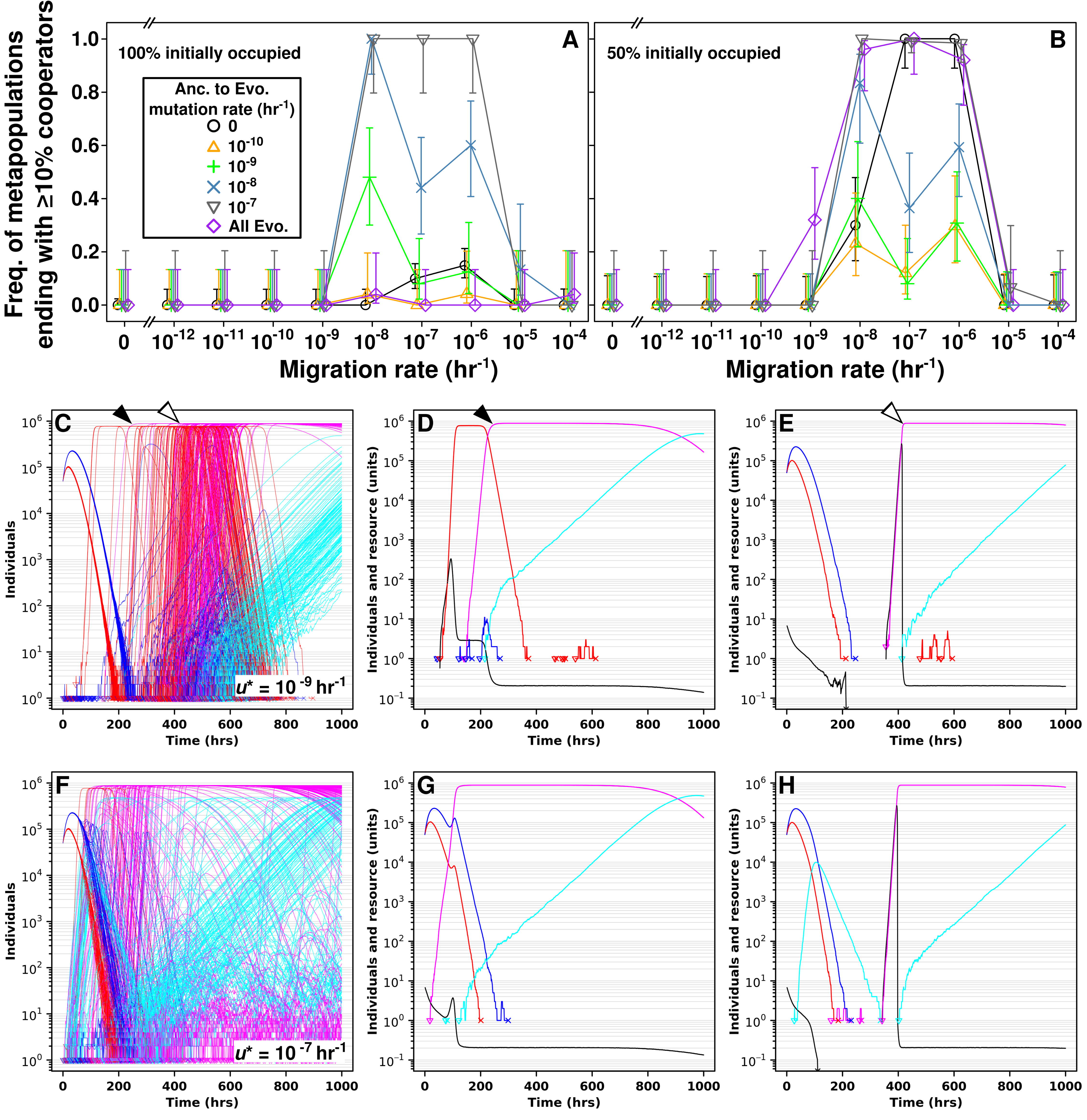

Supplement: S7 Fig — Simulations were initialized with either 100% (A) or 50% (B) of the locations initially occupied. Non-empty locations in each simulation were initialized with a total of 105 cells and a C to D ratio of 1:1. The mutation rate from ancestor to evolved (u*) was varied from 0 (black circle), 10−10 (orange triangle), 10−9 (green plus), 10−8 (blue x), or 10−7 (grey triangle) hr-1. Simulations initiated with 100% evolved (purple diamond) are included for comparison. Data is slightly shifted about the x-axis to aid in visualization of overlapping data points. C-H) Simulation dynamics with 50% of locations initially occupied, a migration rate of 10−7 hr-1, and u* of 10−9 hr-1 (C-E) or 10−7 hr-1 (F-H). C-E) An extremely low mutation rate can hinder the survival of cooperation. C) All 144 locations of a single simulation that eventually went extinct. D-E) Since u* (10−9 hr-1) was very low, ancestor to evolved mutation occurred rarely. At a migration rate of 10−7 hr-1, C dominated the metapopulation (B, black). Thus, initially, C to C* mutation was much more likely to occur than D to D* mutation. Once a C* mutant appeared, it would quickly rise to high frequency (black arrowhead in C and D). Since the migration rate (10−7 hr-1) was much greater than u*, the initial C* population quickly spread throughout the metapopulation (one example is indicated by the white arrowhead in C and E). New migrant C* arrived around the time ancestral types were near carrying capacity, were collapsing, or had gone extinct, which meant that resource was low (e.g., E). This allowed C* to outcompete the remaining ancestor types throughout the metapopulation. However, this coordinated sweep of C* caused a “band” of D* to appear due to mutation. Thus, all locations became synchronized, causing subsequent defector-induced population collapses to occur at similar times, which decreased the chances of cooperator survival compared to moderate u*. (F-H) At a moderate u* (10−7 hr-1), increased heterogeneity [file pcbi.1004645.s007.tiff]
