# Supplementary material for: Defectors Can Create Conditions That Rescue Cooperation
Source: PLoS Comput Biol. 2015 Dec 21;11(12):e1004645. doi: 10.1371/journal.pcbi.1004645 (PMC4687000; doi:10.1371/journal.pcbi.1004645)
Supplement: S1 Appendix — (PDF) [file pcbi.1004645.s008.pdf]

Calculation of the maximum probability of survival of a single cell

Consider an individual *C* cell placed in a location with no resource. We want to estimate Pr(ε), the minimal probability that the entire population goes extinct when its release rate is very high. In general, this will be the probability that the cell does not divide and goes extinct, plus the probability that the cell divides once and both cells go extinct, etc. Let *b* be the number of births before population extinction, then

$$\Pr(\varepsilon)=\Pr(\varepsilon|b=0)+\Pr(\varepsilon|b=1)+\ldots+\Pr(\varepsilon|b=n)\ . \qquad (1)$$

As each term becomes less and less likely to occur, we calculate a lower bound on this probability by only considering the first two terms.

In the first time step Δt, the cell will see no resource, and Pr(ε) is simply the probability of death, p_d. If this cell survives, suppose that it has a probability p_b of giving birth in the next time step. Then in the next time step, the probability of death before cell division becomes $(1\text{-}p_d)(1\text{-}p_b)p_d$. Continuing, we get

$$\Pr(\varepsilon|b=0)\ =\ p_d\left[1+\left[(1-p_d)(1-p_b)\right]^1+\left[(1-p_d)(1-p_b)\right]^2+\ldots+\left[(1-p_d)(1-p_b)\right]^i\right]\ . \qquad (2)$$

Rearranging gives

$$\Pr(\varepsilon|b=0)\ =\ p_d\sum_0^{\infty}\left[(1-p_d)(1-p_b)\right]^i\ =\ \frac{p_d}{p_d+p_b-p_b p_d}\ . \qquad (3)$$

The maximum birth rate was 0.45 hr^{-1}, the death rate was 0.1 hr^{-1}, and the time step was 0.005. Thus, p_b = 0.00225, p_d = 5x10^{-4}, and Pr(ε|*b*=0) = 0.1819. Similarly, we can estimate a lower bound for Pr(ε|*b*=1) using the results for Pr(ε|*b*=0). Let x = Pr(ε|*b*=0), then

$$\Pr(\varepsilon|b=1)\ >\ (1-p_d)\,p_b\,x^2\left[1+\left[(1-p_b)(1-p_d)\right]^1+\ldots+\left[(1-p_b)(1-p_d)\right]^i\right]\ . \qquad (4)$$

Therefore,

$$\Pr(\varepsilon|b=1) > (1-p_d)\,p_b\,x^2 \sum_{i=0}^{\infty}\left[(1-p_b)(1-p_d)\right]^i = \frac{(1-p_d)\,p_b\,x^2}{p_d+p_b-p_b\,p_d}. \qquad (5)$$

Thus, Pr(ε|b=1) > 0.027 and Pr(ε) > 0.2089. The fit in Fig. 2B predicts an ε of ~0.23. Five additional experiments were run with 1024 initial cells and a very high release rate of 10^5 units/hr. The number of extinctions were 226, 229, 223, 234, and 219, for a mean extinction frequency of 0.22 ± 0.005 (2x SEM).
