# Supplementary material for: Defectors Can Create Conditions That Rescue Cooperation
Source: PLoS Comput Biol. 2015 Dec 21;11(12):e1004645. doi: 10.1371/journal.pcbi.1004645 (PMC4687000; doi:10.1371/journal.pcbi.1004645)
Supplement: S2 Appendix — (PDF) [file pcbi.1004645.s009.pdf]

Calculation of the expected slope for growth rate versus release rate

When resource level S reaches a steady state, the population grows at a constant rate. Setting dS/dt=0 in Eq (3), we get $\beta_{ij}(1-N/\kappa)-\gamma\, b_{ij}(S)=0$ for a pure population of ancestral or evolved cooperators. At a population size significantly below the carrying capacity, this becomes $b_{ij}(S)=\beta_{ij}/\gamma$. Thus, the net growth rate is $\beta_{ij}/\gamma-d$. When the net growth rate is plotted against β_{ij} , we obtain a line with slope 1/ γ and intercept -d. Since γ = 5.5 units/cell, the predicted slope is 0.1818 cells per unit of resource, which falls in our 95% CI of 0.180 – 0.182.
